# 4AT screening for delirium in dementia: meta-analysis of diagnostic performance

**DOI:** 10.1590/1980-5764-DN-2025-0378

**Published:** 2025-12-19

**Authors:** Arnold Keane, Karen Elliora Utama, Dhiya Insyira, Abigael Jasmine Angelita, Priyagung Rajab Kuncoro Yekti, Khamelia Malik

**Affiliations:** 1 Universitas Indonesia, Faculty of Medicine, Jakarta, Indonesia.; 2 Ciptomangunkusumo National Hospital, Department of Psychiatry, Jakarta, Indonesia.

**Keywords:** Delirium, Dementia, Aged, Neuropsychological Tests, Delirium, Demência, Idoso, Testes Neuropsicológicos

## Abstract

Delirium is frequently underdiagnosed in older adults, especially those with dementia, due to overlapping clinical features. In this meta-analysis, we evaluate the diagnostic performance of the 4 ‘A’s Test (4AT) in detecting delirium specifically among dementia patients. Five eligible diagnostic accuracy studies were identified in a systematic search in six databases, comprising 1,304 older adults, including 482 with dementia. Pooled sensitivity and specificity were 88 and 79%, respectively, with a diagnostic odds ratio of 32.0, indicating strong discriminatory power. The 4AT high sensitivity supports its use as an initial screening tool to rule out delirium in dementia patients, especially in acute or resource-limited settings. However, moderate specificity highlights the need for confirmatory assessments in positive cases. In this review, we underscore the clinical utility of the 4AT for rapid delirium detection in complex geriatric populations and recommend further research on its performance across dementia subtypes and healthcare environments.

## RESUMO.

Delirium é com frequência subdiagnosticado em idosos, especialmente naqueles com demência, por causa de caraterísticas clínicas sobrepostas. Nesta metanálise, avaliamos o desempenho diagnóstico do teste 4AT na detecção do delirium, especificamente entre pacientes com demência. Cinco estudos de precisão diagnóstica elegíveis foram identificados em uma busca sistemática em seis bases de dados, totalizando 1.304 idosos, 482 com demência. As sensibilidade e especificidade agrupadas foram de 88 e 79%, respectivamente, com um odds ratio diagnóstico de 32, demonstrando alto poder discriminatório. O uso do teste 4AT como uma ferramenta inicial de rastreio para descartar o delirium em pacientes com demência se apoia em sua alta sensibilidade, particularmente em situações de cuidado intensivo ou com limitações de recursos, no entanto destaca-se a necessidade de avaliações confirmatórias em casos positivos em razão da sua especificidade moderada. Nesta revisão, ressaltamos a utilidade clínica do teste 4AT para a detecção rápida de delirium em populações idosas complexas e recomendamos que pesquisas sejam feitas sobre o seu desempenho em subtipos de demência e serviços de saúde.

## INTRODUCTION

 Delirium is an acute neuropsychiatric disorder involving disturbances of consciousness, attention, and cognition, with a typical rapid onset and fluctuating course^1,2^. Delirium is moderately prevalent in the general population (1–2%), but significantly rises in hospitalized older persons, particularly on geriatric wards and in long-stay care institutions, where it occurs up to 40%^3^. Delirium is independently associated with increased morbidity, hospital stay, institutionalization, and mortality^4^. Despite its clinical significance, delirium is frequently underdiagnosed, with rates estimated up to 75% in routine practice^5^. Therefore, early and accurate detection is crucial to improve outcome. 

 The Diagnostic and Statistical Manual of Mental Disorders, Fifth Edition (DSM-V) remains the gold standard for the diagnosis of delirium. However, DSM-based diagnosis requires clinical experience, time, and formal training, therefore being less feasible in settings lacking resources or acute — such as the emergency department^6,7^. The 4 ‘A’s Test (4AT) was developed to address these challenges. As a brief, bedside screening tool, the 4AT screens for alertness assessment at the bedside, orientation using the Abbreviated Mental Test 4 (AMT4), attention task through months of the year backwards, and acute change/fluctuation in mental status. Its brevity (under two minutes) and ease have made it increasingly popular^8-10^. Rapid assessment by trained personnel is also helpful, especially for instruments that can be used by nurses and health professionals other than the physician on duty in the room^11^. 

 The diagnosis of delirium in a patient with preexisting dementia also poses diagnostic challenges, as the clinical presentation may overlap (e.g., shared cognitive symptoms, preexisting cognitive impairment masking acute changes)^8,12^. The presence of overlapping symptoms may weaken the sensitivity and specificity of standard screening tools such as the 4AT. 

 While some researchers have investigated the utility of the 4AT in more general populations of hospitalized patients, a few have focused on its diagnostic accuracy in older people with dementia^12,13^. Authors of some previous meta-analyses also combine data from heterogeneous populations, not presenting findings for dementia subgroups individually, thereby limiting their relevance to clinical practice^10^. In light of the increasing prevalence of dementia-associated comorbidities amidst a global aging population, there is a need to identify screening instruments that can consistently detect superimposed delirium in this vulnerable population^2,4^. Therefore, in this systematic review and meta-analysis, we will establish the diagnostic accuracy of 4AT for detecting delirium in older patients with clinically-established dementia. By synthesizing evidence from diagnostic accuracy studies, we seek to elucidate the strengths and weaknesses of 4AT in this vulnerable population. 

## METHODS

### Study identification

 This systematic review and meta-analysis was conducted in accordance to the Preferred Reporting Items for Systematic Reviews and Meta-Analyses of Diagnostic Test Accuracy (PRISMA-DTA) guidelines and was duly registered in PROSPERO (CRD42025636378)^13^. The literature search was performed on six electronic databases, including PubMed, Cochrane Library, ProQuest, Scopus, Taylor & Francis, and Sage. The search strategy combined terms related to delirium, the 4AT, and diagnostic accuracy, including MeSH terms and Boolean operators as provided in Table 1. Studies published up to October 16, 2024, were considered. 

**Table 1:** Search strategy.

Database	Search Strategy	Hits
PubMed	(((((((((((((delirium[Title/Abstract]) OR (deliri*[Title/Abstract])) OR (acute confusion[Title/Abstract])) OR (acute organic psychosyndrome[Title/Abstract])) OR (acute brain syndrome[Title/Abstract])) OR (acute brain dysfunction[Title/Abstract])) OR (acute brain failure[Title/Abstract])) OR (organic psychosyndrome[Title/Abstract])) OR (metabolic encephalopathy[Title/Abstract])) OR (psycho-organic syndrome[Title/Abstract])) OR (clouded state[Title/Abstract])) OR (clouding of consciousness[Title/Abstract])) OR (Delirium[MeSH Terms])) AND (((4 Assessment Test[Title/Abstract]) OR (4AT[Title/Abstract])) OR (4AT)) AND ((diagnosis[MeSH Terms]) OR (Diagnosis[Title/Abstract] OR Diagnoses[Title/Abstract] OR Diagnose[Title/Abstract] OR Examinations Diagnoses[Title/Abstract] OR Examination Diagnoses[Title/Abstract] OR Examinations[Title/Abstract]))) AND ((((Positive Predictive Value[MeSH Terms]) OR (negative predictive value[MeSH Terms])) OR (Predictive Values Of Tests[Title/Abstract] OR Predictive Value Of Test[Title/Abstract] OR Negative Predictive Value[Title/Abstract] OR Negative Predictive Values[Title/Abstract] OR Predictive Value[Title/Abstract] OR Negative Positive Predictive Value[Title/Abstract] OR Positive Predictive Values[Title/Abstract])) OR ((reliability and validity[MeSH Terms]) OR (Specificity[Title/Abstract] AND Sensitivity[Title/Abstract] OR Sensitivity[Title/Abstract] OR Specificity[Title/Abstract]))))	34
Cochrane	[mh Delirium] OR "acute confusion" OR "acute organic psychosyndrome" OR "acute brain syndrome" OR "acute brain failure" OR "acute brain syndrome" OR "organic psychosyndrome" OR "psycho-organic syndrome" OR "clouded state" OR "clouding of consciousness" AND "4 Assessment Test" OR 4AT AND Diagnose OR Diagnos* OR "Examinations Diagnose" OR "Examination Diagnoses" OR Examinations OR "Negative Predictive Value" OR "negative Predictive Values" OR "Predictive Value" OR "Positive Predictive Values" OR "Positive Predictive Value" OR reliability OR validity OR Specificity OR sensitivity	4
ProQuest	((delirium) AND (4AT) AND (diagnosis) AND (sensitivity OR specificity) AND (systematic review OR Meta-analysis OR meta OR sysrev OR Cross-sectional study) AND (hospitalized) AND (geriatric OR older OR elderly))	59
Scopus	Delirium OR "acute confusion" OR "acute organic psychosyndrome" OR "acute brain syndrome" OR "acute brain failure" OR "acute brain syndrome" OR "organic psychosyndrome" OR "psycho-organic syndrome" OR "clouded state" OR "clouding of consciousness" AND "4 Assessment Test" OR 4AT AND Diagnose OR Diagnos* OR "Examinations Diagnose" OR "Examination Diagnoses" OR Examinations OR "Negative Predictive Value" OR "Negative Predictive Values" OR "Predictive Value" OR "Positive Predictive Values" OR "Positive Predictive Value" OR reliability OR validity OR Specificity OR sensitivity	71
Sage	[All: delirium] AND [All: 4at] AND [All: diagnosis] AND [[All: sensitivity] OR [All: specificity]] AND [All: systematic] AND [[All: review] OR [All: meta-analysis] OR [All: meta] OR [All: sysrev]] AND [All: hospitalized]	10
Taylor & Francis	[All: delirium] AND [All: 4at] AND [All: diagnosis] AND [[All: sensitivity] OR [All: specificity]] AND [All: systematic] AND [[All: review] OR [All: meta-analysis] OR [All: meta] OR [All: sysrev]] AND [All: hospitalized]	12

### Study eligibility criteria

 The inclusion criteria were:

- comparison of the diagnostic accuracy of the 4AT in older adults (≥65 years) with clinically-diagnosed dementia;
- availability of sensitivity and specificity data;
- cross-sectional or prospective study design; and
- reference standard of DSM-IV, DSM-V, or validated structured clinical examination.

The exclusion criteria were studies that did not report on dementia populations, incomplete data, and non-English and non-Indonesian language publications. 

### Study selection

 Five reviewers (AJA, AK, DI, PRKY, and KEU) independently performed study selection and data extraction in Rayyan.ai — without using artificial intelligence (AI) tools to all articles meeting the keywords and the Patient, Intervention, Comparison and Outcome (PICO) criteria across the databases. Disagreements were resolved through consensus. The extracted data were study design, setting, sample size, delirium and dementia prevalence, index test procedures, and diagnostic outcomes (sensitivity, specificity, and likelihood ratios). Values not included in the study were manually estimated in a cautious manner based on the provided information to acquire its derivation. All information was extracted with predefined characteristics. 

### Risk of bias assessment

 Independently, two reviewers (KEU and AJA) evaluated the methodological quality of the included studies using the Quality Assessment of Diagnostic Accuracy Studies (QUADAS-2) tool. Risk of bias was categorized as high, low, or unclear, alongside assessments of applicability concerns, which were narratively summarized. In order to modify the QUADAS-2 tool for the purposes of this review, the threshold item was omitted due to the presence of a predetermined cutoff score (≥4) for delirium identification, as established by the 4AT tool. Furthermore, the time frame between the execution of the index test and the reference standard was standardized to not exceed three hours^10^. 

### Extracted data and synthesis

 In this study, the Cochrane Collaboration’s guidelines for conducting a systematic review were followed, specifically focusing on the evaluation of diagnostic test accuracy (DTA)^14^. To analyze and summarize the diagnostic performance, two hierarchical statistical models were employed: the Hierarchical Summary Receiver Operating Characteristic (HSROC) model and the Bivariate model. Pooled sensitivity and specificity were derived from a bivariate random-effects model. These models allowed for a robust synthesis of data from multiple studies, enhancing the reliability of the findings and facilitating more comprehensive estimations. To further interpret the data, positive and negative likelihood ratios and diagnostic odds ratios were calculated based on the combined sensitivity and specificity values^15^. The analysis included plotting an HSROC curve with 95% confidence and prediction intervals for comprehensive visualization. All statistical analysis was conducted in the R software version 3.2.2 with the "mada" package. 

## RESULTS

### Study identification

**Figure 1:**
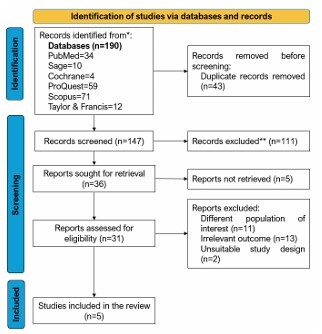
PRISMA flowchart.

Abbreviations: PRISMA, Preferred Reporting Items for Systematic Reviews and Meta-Analyses.

 Of the 190 initial search results, 147 studies remained following deduplication. Following title, abstract, and full-text screening, five studies were eligible. The eligible studies enrolled 1,304 older adults, 482 of whom had a confirmed dementia diagnosis. In Figure 1, we provide a PRISMA flowchart illustrating the study selection process^9,16-19^. 

### Study characteristics

 In Table 2, we describe the characteristics of the five studies included. The proportion of dementia patients varied between 14.3 and 80%, while the study participants ranged from 71 to 392. Moreover, the eligible studies were conducted in five countries in various clinical settings, i.e., emergency departments and geriatric wards. The intervention using 4AT for diagnosing delirium varies between three and 24 hours of admission^9,16-19^. 

**Table 2:** Study characteristics.

Study Author	Country (4AT Language)	Study Design	Setting	Total n	n (%) delirium	n (%) dementia	Mean Age	Reference Standard	Details of 4AT administration
Bellelli et al., 2014^9^	Italy (Italian)	Prospective consecutive study	Acute geriatric and rehabilitation wards	234	29 (12.3)	74 (31.2)	83.9±6.1	Structured standard reference assessment based on DSM-IV-TR by geriatric physician	24 hours upon admission; 15–30 minutes before reference standard by geriatrician (blinded)
O’Sullivan et al., 2017^16^	Ireland (English)	Prospective non-consecutive study	Emergency wards	350	59 (15.2)	82 (21.5)	Median 77	Structured standard reference assessment based on DSM-V by geriatrician	Upon admission and three hours after the first test (blinded)
De et al., 2016^17^	Australia (English)	Prospective consecutive study	Geriatric and orthogeriatric wards	257	159 (62)	205 (80)	86±7.3	Structured standard reference assessment based on DSM-V by consultant geriatrician	Within 72 hours of admission by nurses (blinded)
Pouw et al., 2023^18^	Netherlands (Dutch)	Prospective study	Two geriatric wards and an emergency ward	120	11 (9.1)	16 (13.3)	Median 75	Structured standard reference assessment based on DSM-V by geriatrician	After obtaining verbal consent and reference assessment within four hours of admission (blinded)
Shenkin et al., 2019^19^	United Kingdom (English)	Prospective comparative diagnostic test accuracy study	Geriatric wards, emergency wards, and hospital wards	785	95 (12.1)	71 (14.2)	81.4±6.4	Structured standard reference assessment based on DSM-IV by geriatrician	Within four hours of admission for emergency wards and within 24 hours for acute general wards (blinded)

### Quality assessment

**Figure 2:**
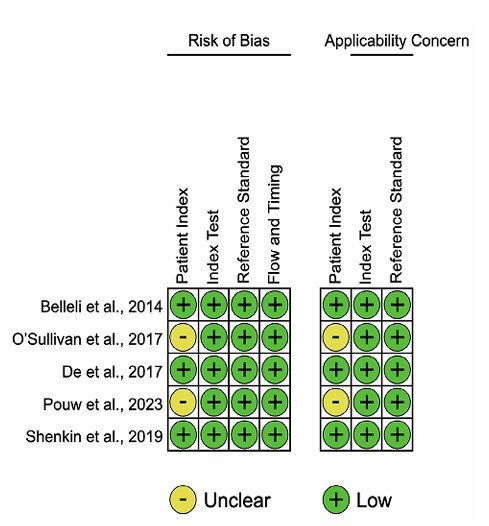
Summary of QUADAS-2 risk of bias and applicability concerns. Abbreviations: QUADAS-2, Quality Assessment of Diagnostic Accuracy Studies.

 All included studies were considered as low or moderate risk of bias according to the QUADAS-2 evaluation (Figure 2). The most common limitation was non-consecutive patient sampling in two studies^16,19^ although consistent inclusion criteria were used and sensitivity analyses were performed. There were no significant concerns regarding applicability. In short, the results confirm that the 4AT is an effective tool for screening for delirium in older adult patients with dementia across different healthcare settings. 

### Diagnostic test accuracy

**Figure 3:**
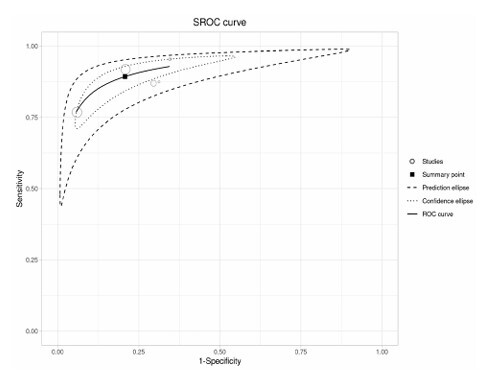
HSROC curve for 4AT for detecting delirium in dementia patients. Abbreviations: HSROC, Hierarchical Summary Receiver Operating Characteristic.

 We verified a pooled sensitivity of 88% (95%CI 81–93) and specificity of 79% (95%CI 65–88) in the 4AT for the diagnosis of delirium in patients with dementia, reflecting good performance of the diagnostic accuracy of the 4AT as a tool to detect delirium in dementia patients (Table 3 and Table 4). In Figure 3, we demonstrate the pooled diagnostic odds ratio, which was 32.0 (95%CI 19.6–52.4), indicating a high level of overall test accuracy. The HSROC curve demonstrated a summary point close to the upper left corner of the graph, thereby indicating its excellent discriminatory power with no significant heterogeneity (I²=0%). 

**Table 3:** Diagnostic values across studies.

Author		Sensitivities (95%CI)	Specificities (95%CI)	PPV	NPV	LR+	LR-	AUROC	Pretest probability✝	Post-test probability✝
Bellelli et al., 2014^9^	Dementia Subgroup	94.1% (91.1–97.1)*	64.9% (58.8–71.0)*	0.444✝	0.974✝	2.682	0.091	0.891	0.29✝	0.523✝
O’Sullivan et al., 2017^16^	Total Sample	89.7% (87.9–92.1)*	84.1% (79.4–88.8)*	0.442✝	0.983✝	5.624	0.123	0.927	0.29✝	0.697✝
De et al., 2016^17^	Dementia	92% (0.79–0.98)	79% (0.64–0.91)	0.82 (0.67–0.92)	0.91 (0.76–0.98)	4.38	0.101	N/A	0.494	0.81✝
Pouw et al., 2023^18^	Total Sample	93% (0.83–0.98)	91% (0.88–0.94)	0.68 (0.57–0.78)	0.99 (0.96–1.00)	10.33	0.076	N/A	N/A	N/A
Shenkin et al., 2019^19^	Suspected Dementia	87% (80–91)	71% (58–82)	87% (80–92)	70% (57–81)	3.00 (2.01–4.43)	0.183✝	0.89	0.697✝	0.873✝
Bellelli et al., 2014^9^	Dementia	88% (0.47–0.99)	69% (0.56–0.80)	0.27	0.98	2.81	0.173✝	0.799	0.112✝	0.257✝
O’Sullivan et al., 2017^16^	Total Sample	76% (61– 87)	94% (92–97)	66% (52–78)	96% (94–98)	12.67✝	0.26✝	0.90 (95%CI 0.84–0.96)	0.143	0.679✝
	Total Sample	87% (81–92)	80% (70–87)	87% (81–92)	80% (69–86)	4.35 (2.9–6.3)	0.162✝	0.92	0.618✝	0.876✝

Abbreviations: PPV, Positive Predictive Value; NPV, Negative Predictive Value; LR+, Positive Likelihood Ratio; LR-, Negative Likelihood Ratio; AUROC, Area Under the Receiving Operating Curve; N/A, not applicable.

Notes: *Confidence interval not provided by the author, hence calculated manually by this paper’s authors; ✝Diagnostic value not provided by the author, hence calculated manually by this paper’s authors.

**Table 4:** Summary of pooled diagnostic accuracy estimates from the bivariate random-effects meta-analysis.

	Estimates
Sensitivity	0.894 (95%CI 0.829–0.936)
Specificity	0.792 (95%CI 0.658–0.883)
DOR	32.033 (95%CI 19.568–52.437)
LR+	4.304 (95%CI 2.6–7.126)
LR-	0.134 (95%CI 0.09–0.201)
Bivariate I^2^	0

Abbreviations: DOR, Diagnostic Odds Ratio; LR+, Positive Likelihood Ratio; LR-, Negative Likelihood Ratio.

Notes: I2, heterogeneity.

## DISCUSSION

### Main findings

 In this meta-analysis, we synthesized data from five diagnostic accuracy studies encompassing 1,304 geriatric patients, 482 of whom had dementia. According to the results, 4AT is an effective tool to screen for delirium in individuals with preexisting dementia, with pooled sensitivity and specificity rates of 88 and 79%, respectively. The high sensitivity supports the usefulness of 4AT as a useful initial screening tool to rule out delirium in negative cases. Moderate specificity indicates that confirmatory testing — especially when the 4AT is positive — is required to eliminate underlying symptoms of dementia from delirium. 

### Contextualization with current literature

 Our findings are in line with and advance the existing meta-analyses evaluating 4AT in older adults more broadly. Tieges et al.^10^ presented pooled sensitivity and specificity estimates of 88%, demonstrating the diagnostic strength of the test. Calf et al.^20^ showed, in another study, that 4AT was very accurate when compared with other assessment tools such as the Confusion Assessment Method (CAM) and the Modified Richmond Agitation-Sedation Scale (mRASS). In contrast, a reduction in sensitivity when applying the 4AT to more heterogeneous clinical groups was verified by Jeong et al.^21^ and Hendry et al.^22^, indicating that routine, less-controlled clinical environments — such as hospital settings — may lower specificity; therefore, the need for subgroup analysis, a section this review particularly explores, was emphasized. We contribute to the literature with our research for specifically targeting the dementia subgroup, a distinction not consistently made by previous meta-analyses. Such specificity increases the clinical applicability of our results to geriatricians and emergency physicians, who are commonly faced with the diagnostic challenge of delirium that occurs in the context of dementia^6,10,23^. 

 The Diagnostic Odds Ratio (DOR) of 32.0 indicates a very good overall discriminatory ability. A positive 4AT test result strongly raises the post-test probability of delirium, and a negative result systematically lowers this probability. Clinicians should, however, be cautious in the interpretation of 4AT results in dementia patients, notably because of the risk of false positives from chronic cognitive impairment^23-25^. In such instances, follow-up evaluation with structured clinical interviews or instruments, such as the CAM, is recommended^19,23^. 

### Strengths and limitations

 This is the first meta-analysis to specifically address the use of 4AT in dementia populations, addressing an important knowledge gap. In this study, we employed rigorous methodology in line with PRISMA-DTA and Cochrane DTA guidelines and novel statistical models (bivariate and HSROC) for data synthesis^14^. However, limitations must be acknowledged. The small number of combined studies (n=5), differences between studies in the prevalence of dementia (14–80%), and application of variable reference standards may restrict generalizability. Publication bias and small-study effects were not formally assessed due to the limited number of included studies, which reduces the power and interpretability of conventional bias diagnostics. This limitation is consistent with recommendations from the Cochrane DTA Handbook^14^. 

### Implications for practice and research

 4AT is an effective delirium screening instrument in individuals with dementia, especially where time, training, and personnel constraints exist. Its high sensitivity renders it a useful tool for ruling out delirium and promoting early treatment^8,24^. A positive 4AT, however, should be considered in the context of clinical assessment and, if possible, complemented by targeted testing. Authors of future studies should investigate the performance of the 4AT between dementia subtypes (e.g., Alzheimer’s disease versus vascular dementia) and across different clinical settings as well as its integration within standard delirium care pathways. 

 All in all, in this meta-analysis we evaluated five studies investigating 4AT as a tool for delirium screening in patients with dementia. According to the findings, 4AT is a highly-sensitive test with an acceptable level of specificity for delirium detection in older adult patients with dementia. Ease of application and lack of extensive training needs make it an appropriate option for screening in acute and resource-limited settings alike. In this review, we validate 4AT use in ongoing delirium screening initiatives and highlight the importance of further validation in other dementia populations and healthcare settings. 

## Data Availability

No new data were generated or analyzed in this study.
